# Development and initial evaluation of the COnfident Parent INternet Guide program for parents of 3–8 year olds

**DOI:** 10.3389/fpsyg.2023.1228144

**Published:** 2023-07-24

**Authors:** Judy Hutchings, Dawn A. Owen, Margiad E. Williams

**Affiliations:** Centre for Evidence Based Early Intervention (CEBEI), College of Human Sciences, Bangor University, Bangor, United Kingdom

**Keywords:** online parenting program, randomized controlled trial, positive parenting, universal provision, feasibility

## Abstract

**Introduction:**

Positive parenting promotes children’s cognitive, social and emotional development and parenting programs based on social learning theory are effective in supporting parents to help reduce behavioral problems among high challenge children. However there is less evidence for programs with non-clinical populations. COPING (COnfident Parent INternet Guide) is a 10-week online universal program for parents of 3 – 8 year olds presenting evidence-informed principles based on social learning theory to support parents in addressing common challenges with their children. This study explored the development and feasibility of delivery of the program in terms of recruitment, retention and acceptability. It also reports on initial program effectiveness, evaluated via a pilot randomized controlled trial.

**Methods:**

Data on child behavior, parental skills and mental health were collected at baseline and three months later for all participants and six months post-baseline for the intervention group only.

**Results:**

Those parents who accessed the course provided very positive feedback however the trial experienced challenges with recruitment and initial engagement, particularly for parents referred by professionals. For parents who engaged with the program there were significant improvements in reported parenting skills with evidence of longer-term maintenance.

**Discussion:**

This paper provides limited evidence of effectiveness for the COPING program however further feasibility work, particularly around recruitment, is needed before conducting larger effectiveness trials.

## 1. Introduction

Good quality parenting establishes positive child behavior and prevents conduct problems. Positive parenting involves giving attention to appropriate behavior, setting clear and consistent boundaries and providing appropriate supervision. Supporting parents to develop these skills has been shown to be helpful in both preventive and treatment trials (Leijten et al., 2019). During the past half-century dramatic lifestyle changes have presented new challenges for all parents and children that generally relate to supervision and boundary setting issues, with everyday challenges around bedtime routines, screen time access and diet that can become increasingly problematic over time (Crnic and Low, 2002; Crnic et al., 2005) that are reported by many parents and summarized below. Consequently it is important that all parents can obtain timely evidence-based advice to address their concerns.

### 1.1. Screen time

One of the biggest concerns for parents is deciding what, and how much, access to the internet and television to allow their children. This was the focus of the UK Chief Medical Officer’s commentary on “Screen based activities and children and young people’s mental health and psychosocial wellbeing: a systematic review” (Davies et al., 2019). The review reports an association between time spent in screen-based activity and increased risk of mental health problems and identifies poor diet, limited physical activity and inadequate sleep as all being impacted by screen time. The Chief Medical Officer recommends that parents set boundaries around online behaviors and also lead by example through not using screens excessively themselves (Davies et al., 2019). Children spend on average 2–5 h per day watching television. More time spent watching television is associated with lower physical and cognitive abilities and greater risk of obesity and mental health issues (Domingues-Montanari, 2017). Excess screen time reduces time spent interacting with others and the amount of conversation that children have is strongly associated with their vocabulary growth, affects their language development (Gridley et al., 2016). Poor language abilities are a growing problem with increasing numbers of children arriving at school with language delay (Action for Children, 2017).

### 1.2. Sleep routines

Children need at least 10 h sleep per night, especially throughout early childhood, with less sleep associated with hyperactivity-impulsivity and lower cognitive performance (Reynaud et al., 2018; Huhdanpää et al., 2019). One third of children aged six or under have a television set in their bedrooms (Helm and Spencer, 2019) and go to bed significantly later than other children (Hale and Guan, 2015). Time spent watching television in bed results in irregular sleep schedules for infants and young children (Brockmann et al., 2016) and compromises learning and attention (Reynaud et al., 2018).

### 1.3. Diet and meal times

In Wales, 26.4% of four to five-year-olds are overweight or obese (Public Health Wales, 2019) and obesity is associated with long lasting negative social and psychological effects (Gibson et al., 2017). Unhealthy foods and sugary drinks, advertised on the television, influence children’s food choices (Kelly et al., 2010). High sugar intake is linked to increased hyperactivity and decreased concentration (Yu et al., 2016), less physical activity and increased television viewing (Kenney and Gortmaker, 2017). A poor quality diet, especially one with predominantly processed food, is associated with poorer academic performance in children (Burrows et al., 2017) with the higher cost of healthy foods, particularly fruits and vegetables, a significant barrier to their consumption (Chapman et al., 2017).

The children of families that regularly eat meals together consume more fruits and vegetables, do better in school, are of average weight, and are less likely to use drugs and alcohol at an early age (Fiese et al., 2012). However, family mealtimes are an increasingly rare occurrence (Jackson et al., 2009).

Evidence-based parenting advice could benefit a broad group of parents, improve the well-being of all children and prevent the subsequent risk of mental health problems (Sherr et al., 2014). Advantages of universal provision include (1) support for parents who want to provide their children with the best outcomes (Ulfsdotter et al., 2014), (2) access to evidence-based information for parents based on core social learning theory principles to address common parenting challenges.

Early universal trials, incorporating the same theoretical underpinnings as targeted/preventive programs have recruited varied samples of parents and shown promise (Reedtz et al., 2011). However, particularly given the challenges of the COVID 19 pandemic, more research is needed on accessible ways of providing parents with appropriate information.

Internet enabled technology is now widely available, with 96% of UK homes having access (Livingstone et al., 2011; Office for National Statistics, 2020). It has become the fastest growing resource for parents with many using the web for parenting support and advice on a daily basis (Dworkin et al., 2013). The parenting website “Mumsnet,” the UK’s busiest social network for parents, receives almost 10 million visits every month (Mumsnet, 2019). However, online advice, that relies on parental suggestions and past experiences shared through discussion boards (Wald et al., 2007) is of uncertain validity.

Since many parents now seek advice on parenting through the internet, this offers the potential to provide evidence-based support that will help to address the new and widespread challenges faced by many parents. It has flexibility so can fit around work and other schedules and childcare activities and offers a mode of advice that is efficient, accessible and convenient for large numbers of parents (Breitenstein and Gross, 2013). Furthermore some parents who have used it report a preference for interventions delivered using technology (Metzler et al., 2012). Support based on the core principles of social learning theory has demonstrated effectiveness in increasing positive parenting from universal prevention trials (Leijten et al., 2019) and now needs to be made universally available to parents.

Hansen et al. (2019) conducted a systematic review of technology-assisted parenting programs and identified 25 interventions, of which only six were universal. Other systematic reviews have shown online parenting programs to be effective in improving parenting skills as well as reducing child behavior problems (e.g., Florean et al., 2020; Spencer et al., 2020; Thongseiratch et al., 2020), however they predominantly targeted parents of children with significant behavioral problems and frequently had additional components, such as weekly phone calls.

The current study reports on feasibility questions around recruitment to, intervention delivery, study retention, and acceptability of the COPING (COnfident Parent INternet Guide) online program that was developed for parents of children aged 3–8 years who wanted to learn more about positive parenting. It also reports on the recruitment and personal characteristics of the recruited parents and initial evidence of effectiveness for those parents who engaged with the program.

## 2. Materials and methods

### 2.1. Participants

The primary caregivers of a child aged 3–8 years who expressed an interest in learning more about positive parenting were invited to enroll. Participants had to have access to the internet via a tablet, PC or laptop and a good understanding of English. The recruitment leaflet described COPING as a universal program for people wanting to learn more about positive parenting. Due to time constraints, this was part of a PhD study, recruitment was undertaken in two ways: (i) posters were distributed to local primary schools and nurseries; (ii) health visitors, school nurses and three other professionals working with children distributed leaflets to families. Health visitors are the only UK profession that have all pre-school children on their caseloads. Interested parents were asked to provide verbal consent for their contact details to be forwarded to the research team or to contact the research team directly using the details from the poster. A member of the research team then arranged a home-visit to discuss the study further and answer questions. If parents were happy to proceed, written informed consent was obtained. Once obtained, parents were asked to complete baseline measures.

Ethical approval was granted by NHS Betsi Cadwaladr University Health Board (REC 15/WA/0463) and Bangor University Psychology Ethics Committee (ref: 2015-15506). All participating parents provided written informed consent.

### 2.2. Measures

#### 2.2.1. Family demographics

A baseline family demographics questionnaire included information on marital status, parent education, employment status, child age and parent age at birth of first child.

#### 2.2.2. Feasibility outcomes

Feasibility was operationalized in terms of recruitment, retention and acceptability (engagement and satisfaction). For satisfaction, parents in the intervention condition completed a questionnaire that consisted of two yes/no questions about the usefulness of the program and whether they would recommend it to other parents, and two open-ended questions exploring aspects of the program that they liked and aspects that they would change.

#### 2.2.3. Parenting skills

Categories from the Dyadic Parent-Child Interaction Coding System (DPICS; Robinson and Eyberg, 1981) were used to assess parenting skills in an observed parent–child interaction task. The DPICS was designed to assess the quality of parent–child interaction and has high inter-rater reliability for both parent and child behaviors (Robinson and Eyberg, 1981). The DPICS has been extensively used in parenting research (Hutchings et al., 2007; Williams et al., 2020). The following skills were coded: praise, direct (clear) commands, indirect (vague) commands, questions, and negative parenting (comprised of negative commands and critical statements). Self-reported parenting skills were assessed with the Parenting Scale (PS; Arnold et al., 1993), a 30-item parent report inventory measuring parenting practices. The internal consistency for the PS total in the current study was α = 0.74–0.84. Parental competence was measured using the Parental Sense of Competence (PSoC; Johnston and Mash, 1989) questionnaire, a 17-item parent report measure assessing parental competence on two dimensions: satisfaction and efficacy. Satisfaction questions measure parental anxiety, motivation and frustration, and efficacy questions measure parental competence, capability and problem-solving abilities. The internal consistency of the PSoC in the current study were as follows: Efficacy (α = 0.66–0.82); Satisfaction (α = 0.57–0.68).

#### 2.2.4. Child behavior

The Eyberg Child Behavior Inventory (ECBI; Eyberg and Robison, 1983) measures the frequency and intensity of behavioral problems in children aged 2–16 years and was used to assess the extent of child conduct problems. This 36-item measure has two sub-scales (1) intensity and (2) problem. Intensity sub-scale responses are recorded on a 7-point scale, from 1 (never) to 7 (always). Problem subscale responses consists of “*Yes*” or “*No*” and are scored by summing the number of “*Yes*” responses to the question “*Is this a problem for you?*” Internal consistency for the ECBI in the present trial was as follows: Intensity (α = 0.90–0.94); Problem (α = 0.90–0.94).

#### 2.2.5. Parental mental health

The General Health Questionnaire (GHQ; Goldberg, 1978), a 30-item parent report, is a screening tool measuring parental mental health in response to questions including “*been feeling hopeful about your own future?*” and “*been feeling unhappy or depressed*?” Internal consistency for the GHQ in the present study ranged from 0.92 to 0.96.

### 2.3. Procedures

#### 2.3.1. Data collection

Home visits were conducted with each participating family to collect baseline and three-month follow-up measures as well as six-month follow-up measures with intervention parents only. Visits lasted for approximately 1 h (30 min for the questionnaires and 30 min for the observation). Primary caregivers were asked to play with their child as they normally would for 30 min. Observations were live coded by one of two trained coders blind to participant group allocation. Both coders were DPICS trained and coded videos together until 80% agreement for each category was achieved prior to undertaking observations for the trial. Parent-child dyads were observed for 30 min at each time point. Inter-rater reliability was undertaken for at least 20% of all observations at all three-time points (baseline = 21.4%; 3-month follow-up = 22.2%; 6-month follow-up = 20%). Intraclass correlations (ICCs) were high across all time-points (ICCs = 0.95–0.99). At the end of the initial data collection the wait-list control process was explained and the researcher discussed means of accessing the program to ensure that this was technically feasible for the parent.

#### 2.3.2. Randomization

After informed consent was obtained and baseline measures collected, parents were randomly allocated to intervention or three-month wait-list control, on a 2:1 ratio stratified by child age and gender. The Centre administrator undertook randomization using the online software “sealed envelope” to ensure that data collectors remained blind to group allocation.

### 2.4. Intervention

The COPING (COnfident Parent INternet Guide) program was developed and initially tested in a feasibility study with 20 parents (Owen and Hutchings, 2017), 19 of whom were recruited via existing networks and one via a leaflet in a local nursery. Feedback on usefulness and acceptability resulted in modification for the current trial, included adaptations to enable access by tablet users, an option to look back over previously completed chapters, the inclusion of more video examples of positive parenting and text message prompting as reminders to log on to address attrition challenges.

The program is based on a book of evidence-based advice for parents (Hutchings, 2015; Hutchings, 2019). It summarizes key parenting skills and introduces behavioral principles associated with good child outcomes. COPING contains topics to strengthen positive parenting behaviors by encouraging parents to be positive role models, to praise and reward desirable child behavior, to give clear instructions, ignore child protests and manage resistance. It also has sessions on teaching skills and on promoting children’s language (Hutchings, 2019). It has eight content and two revision chapters. Parents are asked to log in and complete one chapter each week. Each chapter presents information, videos of positive parenting strategies demonstrating the skills introduced each week, questions based on the videos and multiple-choice quizzes with feedback. Parents are encouraged to practice the skills at home with their child and each chapter concludes with a suggested activity for the week, for example, “*praise your child after they have done something good this week*”. The program delivers behavioral principles using the same evidence-based principles in program delivery (feedback, video modeling, etc.). For a detailed description of the program see Hutchings et al. (2018).

The program was created using the LifeGuide software developed at the University of Southampton, a cost-efficient set of tools to deliver and evaluate online behavior change interventions (Hare et al., 2009). LifeGuide allows researchers to use behavioral principles in both program content (Hare et al., 2009; Yardley et al., 2009) and delivery (feedback, text message prompts, video examples, etc.).

The program was set up to provide direct feedback to the researchers on participant use and also to provide prompts to participants to engage in the next session after 1 week. It was not possible to access the next session until a week had passed since completing the previous one to allow time to practice the skills presented in the session.

### 2.5. Data analysis

#### 2.5.1. Quantitative

The target sample size was 60 participants (40 intervention and 20 control) based on recommendations suggesting that feasibility trials include a sufficient sample to answer feasibility questions (National Institute for Health Research, 2013) and as planned in the registered protocol paper (Owen et al., 2018). Baseline characteristics, the impact of recruitment source and qualitative parent feedback data are reported as part of the feasibility outcomes. Outcome data were analyzed using SPSS version 25. For initial effectiveness, variable residuals for outcomes were examined using Q-Q plots to detect any violations in normality. Square-root transformations were used to normalized the observed variables (and used in the analyses) but this was not possible for the GHQ and observed negative parenting. The primary analyses consisted of repeated measures ANOVA models with time as the within-group variable and condition as the between-group variable using complete case data. Model estimates for both time and condition and 95% confidence intervals are reported as well as effect sizes with 95% confidence intervals. Effect sizes were calculated by dividing the model estimates for the effect of condition on each outcome by its pooled baseline standard deviation. Interpretation of the effect sizes was based on Cohen’s *d* values (Cohen, 1988). For the GHQ and observed negative parenting variables, non-parametric Mann-Whitney U Tests were run. Long-term maintenance exploration involved paired *t*-tests and confidence intervals were examined to assess the difference between baseline, three-month and six-month outcomes for the intervention sample only.

#### 2.5.2. Qualitative

Responses to the open questions on the satisfaction measure were analyzed using Braun and Clarke’s (2006) thematic analysis strategy. Codes were generated inductively through reading, coding, re-reading and re-coding to enable responses to be grouped together into themes. This process was undertaken by an independent researcher and checked for accuracy by a second coder.

## 3. Results

### 3.1. Recruitment

**Figure 1:**
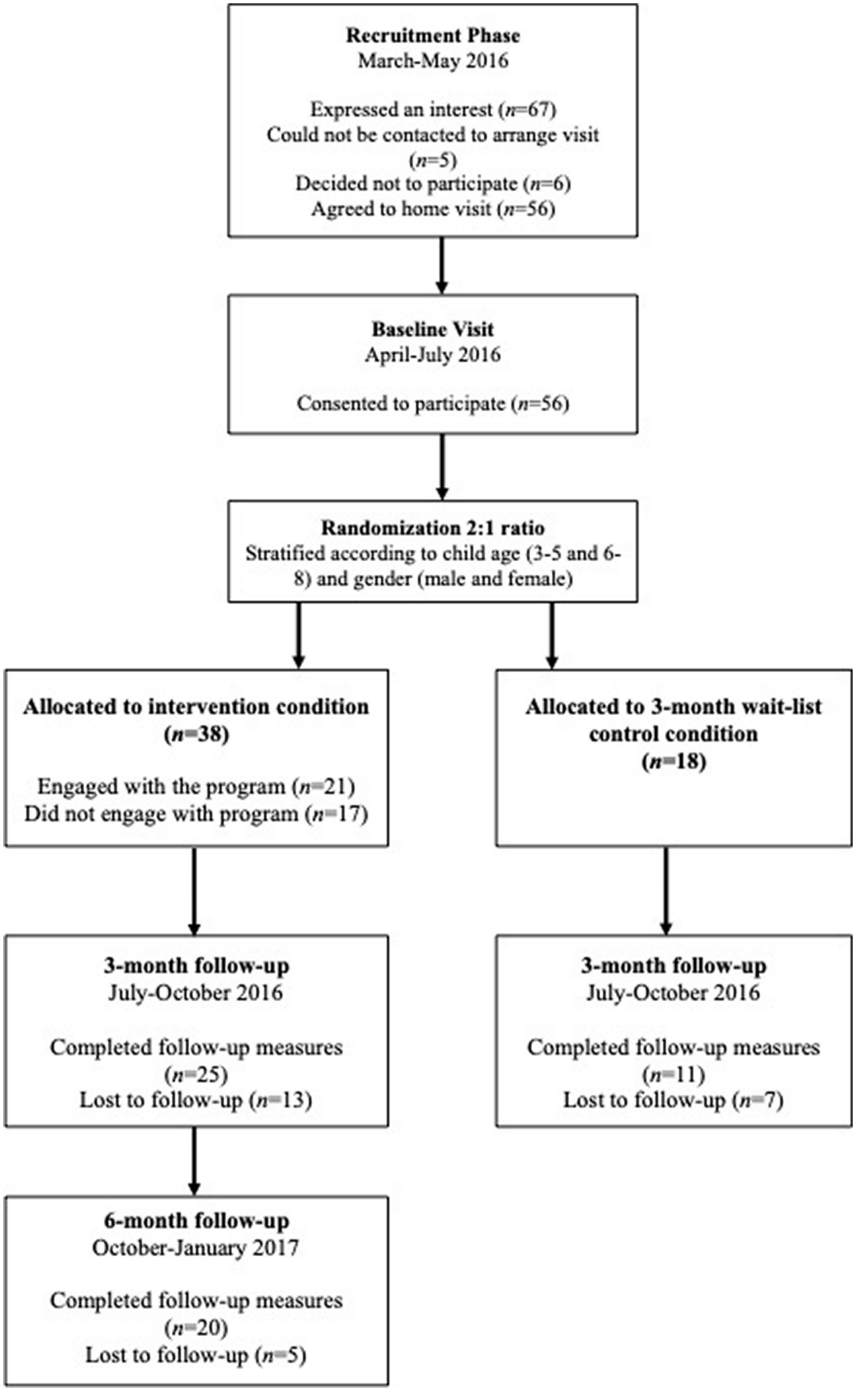
Participant flow diagram.

Sixty-seven parents expressed an interest in participating of whom fifty-six (83.6%) were recruited into the study between March and July 2016. Twenty-one parents were recruited via posters distributed in schools and nurseries (37.5%) whilst 35 were informed about the study via a health care professional (e.g., health visitor) (Figure 1).

#### 3.1.1. Sample characteristics

Demographic characteristics are presented in Table 1 which also shows the characteristics by source of recruitment, leaflets distributed in schools/nurseries or via HVs etc. The mean child age was 57.38 months (SD = 19.12) with over 70% being male. All but one primary carer was female. Thirty-eight families were randomized to intervention and 18 to the waitlist control condition. There were no significant differences between intervention and control parents on any demographic or baseline measures.

**Table 1:** Participant demographics.

Family characteristics	All (*N* = 56)	Recruited via poster (*n* = 21)	Recruited via professional (*n* = 35)
Child gender, male: *n* (%)	40 (71.4)	18 (85.7)	22 (62.9)
Child age, months: *M* (SD)	57.38 (19.12)	52.62 (19.38)	60.23 (18.65)
Parent gender, female: *n* (%)	55 (98.2)	20 (95.2)	35 (100)
Parent age, years: *M* (SD)	33.59 (6.67)	36.33 (5.30)	31.94 (6.93)*
Parent age birth first child, years: *M* (SD)	26.16 (5.97)	30.00 (5.04)	23.79 (5.27)**
Post 16 education: *n* (%)	42 (75.0)	18 (85.7)	24 (68.6)
Married/cohabiting: *n* (%)	46 (82.1)	19 (90.5)	27 (77.1)
Employment: *n* (%)	50 (89.3)	19 (90.5)	31 (88.6)
Clinical cut-off ECBI-I (131), above: *n* (%)	25 (44.6)	6 (28.6)	19 (54.3)
Clinical cut-off ECBI-P (15), above: *n* (%)	27 (48.2)	7 (33.3)	20 (57.1)
Clinical cut-off GHQ, above: *n* (%)	25 (44.6)	6 (28.6)	19 (54.3)

*p < 0.05; **p < 0.001.

There were significant differences based on the source of recruitment to the study (see Tables 1, 2). Parents who were referred to the study by a health care professional reported significantly higher ratings of child behavior problems (Mean = 142.03 vs. 120.19; *p* = 0.006), were significantly younger at baseline (Mean = 31.94 vs. 36.33, *p* = 0.016), and were significantly younger at the birth of their first child (Mean = 23.79 vs. 30.00, *p* ≤ 0.001) than parents who self-referred using a poster/leaflet. Parents recruited via posters used significantly more direct (clear) commands (Median = 6.00 vs. 4.00, *p* = 0.008) and more negative parenting (Median = 14.50 vs. 6.00, *p* ≤ 0.001).

**Table 2:** Baseline characteristics.

Baseline measures	All (*N* = 56)	Recruited via poster (*n* = 21)	Recruited via professional (*n* = 35)
	*M* (SD)	*M* (SD)	*M* (SD)
ECBI intensity	133.84 (30.118)	120.19 (26.32)	142.03 (29.59)*
PS total	3.18 (0.51)	3.11 (0.57)	3.22 (0.48)
PSOC efficacy	23.95 (3.51)	23.33 (3.81)	24.31 (3.23)
PSOC satisfaction	29.93 (4.89)	31.14 (3.79)	29.30 (5.37)
	**Median (range)**	**Median (range)**	**Median (range)**
ECBI problem (cut-off 15)	12.00 (0–33)	9.00 (1–21)	16.00 (0–33)
GHQ	2.00 (0–23)	1.00 (0–18)	4.50 (0–23)
Observed direct commands	6.10 (0–37)	6.00 (0–37)	4.00 (0–9)*
Observed indirect commands	38.00 (5–98)	39.50 (6–98)	36.00 (5–79)
Observed questions	74.00 (3–192)	71.50 (3–192)	83.00 (3–181)
Observed praise	9.00 (1–34)	9.00 (1–34)	9.00 (1–29)
Observed negative parenting	11.00 (0–39)	14.50 (0–39)	6.0 (0–20)**

*p* < 0.05; **p < 0.001.

### 3.2. Acceptability

#### 3.2.1. Engagement

Of the 38 parents randomized to the intervention condition, 21 (55.3%) accessed the program and 17 (44.7%) did not engage at all. Parents who did not engage had significantly higher median baseline (GHQ) mental health problems (4.00 vs. 1.00; *p* = 0.036) and were younger when they had their first child (Mean years = 23.71 vs. 28.50; *p* = 0.027). Mean chapter completion was two (*M* = 2.03, SD = 3.10, range = 0–10).

#### 3.2.2. Satisfaction

Intervention families were asked to complete a short feedback form following completion of the three-month follow-up measures. Of the 21 parents who engaged with the program, 17 provided follow-up data of whom 13 (76.5%) completed the feedback form (with four participants reporting that they had not completed enough chapters to provide feedback).

The 13 parents who completed the participant feedback form reported mean session attendance of 4.5 sessions, with several indicating their intention to continue with the program. All 13 responded yes to the question did you find the program useful and said that they would recommend it to others.

A total of 48 comments (mean of 3.46 per participant) were coded and grouped into six summary themes (see Table 3). The most prominent theme was about the usefulness of the program and the different components. Parents reported finding “the whole [program] really useful” and that they had “enjoyed this program.” Others mentioned the usefulness of different components to the program including the summaries of previous content at the start of each chapter and the full revision chapters mid-way through and at the end of the program. Some commented on the usefulness of the videos as they showed examples of the skills and depicted children of a similar age to their own. Others mentioned the usefulness of being able to access the program at their own time and pace. Three comments mentioned the quizzes as being particularly useful.

**Table 3:** Themes from the satisfaction questionnaire.

Theme	Number of responses
Usefulness	19
Ease of program	12
Changes in own behavior	10
Positive impact on child/family	4
Software challenges	3

Another theme identified was about the accessibility of the program with parents reporting positively on the layout and language. Parents said they “liked how the program was broken down into manageable step-by-step instructions” and highlighted that the program was “easy to understand and follow.” Another theme related to changes in the parents’ own behavior and how these then impact on their child’s behavior. One parent said: “it’s been really interesting to learn how significant my behavior as a parent is and its impact on my child’s behavior.” Parent reported feeling “real confidence” in their skills and in how they deal with daily challenges. There was also a theme about the impact on the child. Parents reported that their child’s behavior had “greatly improved.” Finally, parents were reporting a barrier to the program in terms of the software challenges.

### 3.3. Retention

Twenty parents (35.7%) were lost to the three-month follow-up, 13 from the intervention and seven from the control condition. Of the 13 intervention parents lost at follow-up, nine (69.2%) had not completed any chapters and four (30.8%) had completed only one chapter.

Independent *t*-tests and chi-square analyses showed a significant difference between participants and those seen at follow-up with those lost to the three-month follow-up having been more likely to have been recruited to the study via a professional (*n* = 16 vs. 4; *p* = 0.044) and less likely to engage in the program (Median chapters completed (range) = 0.00 (0–1) vs. 1.00 (0–10); *p* = 0.010). They were also younger at the birth of their first child (Mean years = 22.90 vs. 28.03; *p* = 0.001) and used more indirect commands at baseline [Median (range) = 44.00 (18–84) vs. 34.00 (5–98); *p* = 0.039].

At the six-month follow-up of intervention parents only, 18 (47.4%) parents were lost. Of these, 12 (66.7%) had not completed any chapters, five (27.8%) had completed one chapter and one (5.6%) had completed two chapters. As with the initial follow-up, independent *t*-tests and chi-square analyses again showed a significant difference with parents lost to follow-up having been recruited via a professional (*n* = 14 vs. 4; *p* = 0.039), less likely to engage in the program [Median chapters completed (range) = 0.00 (0–2) vs. 1.50 (0–10); *p* = 0.002], younger (Mean years = 31.67 vs. 36.35; *p* = 0.043) and younger at the birth of their first child (Mean years = 23.61 vs. 28.84; *p* = 0.012).

### 3.4. Initial effectiveness

**Figure 2:**
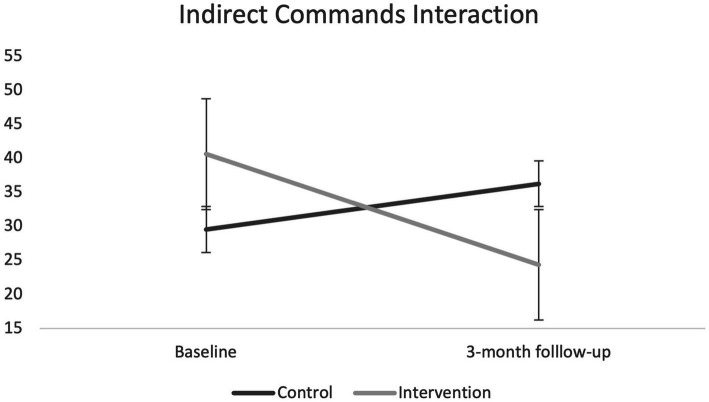
Graph of significant interaction for indirect (vague) commands.

Raw descriptive statistics for the outcomes are shown in Table 4. There was a significant interaction for observed indirect commands (*p* = 0.009) showing that parents in the intervention condition reduced the number of indirect commands between baseline and follow-up whilst parents in the control condition increased their number of indirect commands (see Figure 2).

**Table 4:** Descriptive statistics and RM ANOVA results for intervention and control.

	Control (*n* = 11)		Intervention (*n* = 25)		Estimate (95% CI)	Effects size (95% CI)
	Baseline	3-month FU	Baseline	3-month FU		
	*M* (SD)	*M* (SD)	*M* (SD)	*M* (SD)		
ECBI-I	136.00 (32.89)	124.73 (33.39)	131.24 (33.16)	118.64 (31.01)	−5.42 (−28.24, 17.40)	−0.16 (−0.85, 0.52)
ECBI-P	14.91 (8.14)	13.36 (9.56)	12.32 (7.94)	9.20 (8.62)	−0.33 (−0.91, 0.25)	−0.46 (−1.27, 0.35)
PS-T	3.15 (0.42)	2.95 (0.48)	3.27 (0.54)	2.99 (0.65)	−0.08 (−0.45, 0.29)	−0.17 (−0.94, 0.60)
PSOC-E	23.18 (3.82)	23.45 (3.53)	23.72 (3.53)	24.24 (3.88)	0.66 (−1.74, 3.06)	0.18 (−0.47, 0.83)
PSOC-S	31.45 (3.93)	30.90 (4.35)	28.96 (4.63)	30.04 (3.98)	1.68 (−0.92, 4.29)	0.39 (−0.21, 1.00)
DC^a^	4.82 (4.22)	3.09 (4.46)	6.48 (4.76)	4.64 (4.43)	−0.47 (−1.06, 0.12)	−0.47 (−1.08, 0.12)
IC^a^	29.55 (24.87)	36.27 (21.92)	40.64 (21.70)	24.36 (15.24)	−0.01 (−1.04, 1.03)^§^	−0.01 (−0.54, 0.53)
Question^a^	73.27 (50.56)	50.82 (33.99)	77.96 (46.96)	59.28 (30.31)	−6.58 (−30.92, 17.77)	0.14 (−0.65, 0.37)
Praise^a^	9.27 (8.78)	11.18 (15.20)	9.28 (5.76)	15.44 (12.72)	0.55 (−0.39, 1.50)	0.49 (−0.35, 1.32)
	**Median (range)**	**Median (range)**	**Median (range)**	**Median (range)**	***Z***	**Effect size**
GHQ	1.00 (0–13)	1.00 (0–11)	2.00 (0–23)	2.00 (0–29)	−0.83	0.14
NP^a^	9.00 (0–26)	6.00 (0–29)	10.00 (1–39)	3.00 (0–30)	−0.62	0.10

a Observed outcomes. DC, Direct command; ECBI-I, Eyberg Child Behavior Inventory Intensity scale; ECBI-P, Eyberg Child Behavior Inventory Problem scale; GHQ, General Health Questionnaire; IC, Indirect command; NP, Negative parenting; PS-T, Parenting Scale Total score; PSOC-E, Parenting Sense of Competence Efficacy score; PSOC-S, Parenting Sense of Competence Satisfaction score.

§ Significant interaction.

#### 3.4.1. Longer-term maintenance

Two comparisons were conducted to explore potential maintenance effects, one between baseline and six-month follow-up and one between three-month and six-month follow-up for the 15 parents who had engaged in the intervention (see Table 5). In the comparison from baseline to six-months, parents reported significant improvements in child behavior as well as measures of parenting competence and skills. In the three-month to six-month comparison, observed parent praise showed a significant improvement.

**Table 5:** Long-term maintenance outcomes at 3-months and 6-months post-intervention (*n* = 15).

	BL	3-month FU	6-month FU	BL to 6-month FU	3- to 6-month FU
	*M* (SD)	*M* (SD)	*M* (SD)	Mean difference (95% CI)	Mean difference (95% CI)
ECBI intensity	130.87 (33.00)	118.07 (32.47)	116.80 (32.90)	−14.07 (−24.30, −3.84)*	−1.27 (−11.04, 8.51)
PS total	3.23 (0.57)	2.90 (0.63)	2.73 (0.61)	−0.50 (−0.78, −0.23)*	−0.17 (−0.40, 0.06)
PSOC efficacy	23.27 (3.24)	25.13 (3.56)	25.67 (3.96)	2.40 (0.17, 4.63)*	0.53 (−1.15, 2.22)
PSOC satisfaction	28.87 (4.02)	30.80 (3.88)	32.07 (4.06)	3.20 (0.45, 5.95)*	1.27 (−0.95, 3.49)
Direct command^a^	5.27 (2.87)	5.00 (4.80)	8.00 (6.72)	0.19 (−0.62, 0.99)	0.48 (−0.23, 1.19)
Indirect command^a^	43.87 (22.29)	25.33 (15.93)	25.13 (9.08)	−1.54 (−2.36, −0.73)*	−0.11 (−0.79, 0.57)
Question^a^	90.33 (52.38)	71.20 (28.24)	72.60 (31.35)	−0.81 (−2.20, 0.58)	0.05 (−0.81, 0.90)
Praise^a^	9.60 (5.74)	18.47 (11.74)	25.00 (12.20)	1.89 (1.43, 2.35)*	0.71 (0.11, 1.31)*
Negative parenting^a^	10.53 (9.25)	4.87 (6.26)	3.73 (2.92)	−1.21 (−1.89, −0.54)	−0.08 (−0.73, 0.58)
	**Median (range)**	**Median (range)**	**Median (range)**	***Z (p)***	***Z (p)***
ECBI problem	10.00 (3–27)	6.00 (0–24)	3.00 (0–27)	−2.52 (0.012)	−1.15 (0.252)
GHQ	1.00 (0–19)	0.00 (0–6)	0.00 (0–7)	−1.21 (0.227)	−0.12 (0.905)

a Observed outcomes. * CIs do not cross zero. BL, baseline; FU, follow-up; CI, confidence interval; ECBI, Eyberg Child Behavior Inventory; GHQ, General Health Questionnaire.

## 4. Discussion

The COVID-19 pandemic has been challenging for all parents, increasing concerns about how to deal with everyday challenges particularly around screen time and sleep routines and highlighting the need to develop universal parenting support. Positive parenting strategies are key to ensuring good outcomes for all children (Gardner et al., 2006; Hutchings et al., 2007) however, most parenting programs, including online programs, are targeted at clinically referred or high-risk children (Hutchings et al., 2007). Parents are increasingly turning to the internet for advice and the COPING on-line program was designed to target a universal population for parents of 3–8 year-olds with no additional support other than prompt text message reminders to log on that, unfortunately due to technical problems with the LifeGuide software, did not occur.

The trial examined the feasibility, in terms of recruitment and delivery and acceptability, as well as the initial effectiveness, of the intervention, developed as a universal program. Feasibility analysis showed that, despite the recruitment literature clearly describing the goals of the program as targeting everyday parent challenges, recruitment via professionals, predominantly health visitors, was less successful in both retention and outcomes than that achieved by parents recruited via leaflets in schools and nurseries. Professionals recruited parents who were experiencing more challenges and who were less likely to engage in, or complete, the program or provide follow-up data than those who self-recruited from poster distribution through nurseries and primary schools. In this trial therefore, without additional support, the program was relatively unsuccessful for parents recruited by professionals and experiencing significant levels of mental health difficulties and child behavior problems. Recruitment through posters on the other hand was more successful in enrolling parents many of whom engaged with, and benefitted from, the program. Growing pressures on child professional services suggests that health visitors and other professionals encouraged families with significant challenges to engage due to their own work pressures but the evidence from this trial suggests that, as delivered, COPING was insufficient for the perceived needs of these families. Without additional support, online interventions are unlikely to be suitable for all families (Calam et al., 2008), suggesting that additional strategies are needed (e.g., telephone support and/or text reminders) or more targeted interventions offered to this higher risk population.

Attrition rates were high at both the 3- and 6-month follow-ups, however they are consistent with other studies (Enebrink et al., 2012; Chacko et al., 2016) and have been problematic in other web-based parenting interventions (Sanders et al., 2008, 2012). There were also very variable rates of intervention engagement with the mean number of chapters completed being two. Parents who did not engage at all were more likely to have poorer mental health, similar to the findings of Dadds et al. (2019), suggesting the need for additional support for successful engagement. Program features such as telephone or email prompts has been shown to be a core component for successful online parenting programs (Thongseiratch et al., 2020). Although the COPING intervention was programed to send reminder texts, for technical reasons caused by failure of the LifeGuide operating system this did not happen and may have contributed to the poor engagement rates.

In terms of initial effectiveness, results were limited although parents who engaged with the programs reported some positive outcomes. Complete case analyses showed a significant interaction between intervention and control for indirect commands at the three-month follow-up. No other outcome showed significant differences. At the six-month follow-up, intervention parents who had engaged in the program showed improvements in child behavior and parenting skills when compared to baseline. All results should be interpreted with caution given the lack of statistical power to detect significant differences and no comparison group at the six-month follow-up.

Of those who did engage, 13 completed a satisfaction questionnaire reporting that they would recommend it to other parents as well as positive comments about different aspects of the program such as changes in their own parenting behavior, that the content was easy to understand, useful and convenient and that it had positive impact on their children. Several parents reported a barrier to accessing the program due to software challenges which impacted on the engagement statistics.

### 4.1. Limitations

High attrition presents the biggest limitation in any trial as it points to a risk of bias in reporting outcomes. However this was a feasibility trial and the data suggest that for those who did access the program there were some positive outcomes and, given that the attrition demonstrated a population for whom the program was not successful, those with children with significant child behavioral problems, this was an important feasibility finding. Furthermore given that this was a feasibility trial with a small sample the statistical analysis was exploratory and any attempt to impute or undertake other statistical procedures on this sample would be inappropriate.

The initial trial (Owen and Hutchings, 2017) did not address recruitment and although the program was significantly adapted following the earlier feasibility trial, these adaptations were primarily focused on content and, as demonstrated by the findings, recruitment and access need significant attention in a future trial.

Technical difficulties meant that the planned text message system was not delivered and no additional support was provided. Some parents also reported problems with the software in accessing the program which is something that is being addressing in a current trial. The use of an online learning management system may be more appropriate as they include many additional features such as notifications for parents for prompting engagement and less access restrictions.

Another limitation was that the program gave access to subsequent chapters only after 1 week had elapsed. It was also not possible for parents to choose which chapters to complete, having to complete each chapter in succession. Current research is exploring the use of content delivered at the pace of the parent and in which parents can choose which chapters are more applicable to them and the challenges that they are currently managing. Finally, due to funding/time restrictions only 56 families were recruited and it was not possible to obtain follow-up data beyond 6 months.

## 5. Conclusion

It is important to provide universal, easily available, access to evidence-based parenting skills. For those who accessed the program it significantly improved aspects of both parenting and child behavior at six-months, albeit with no control group comparison, and achieved high satisfaction ratings. The feasibility of delivery of the COPING parent online universal program was established however the issue of recruiting those for whom it would be useful needs further consideration and could include newspaper adverts and advertising through existing websites such as Mumsnet.

The trial demonstrated positive outcomes for the targeted low-risk population but was less good at engaging or retaining those who were reporting significant child behavior problems. This suggests that retention of parents reporting significant problems needs a more scaffolded program and highlights aspects of program delivery that we are currently addressing in order to recruit participation from parents with everyday concerns about parenting who otherwise might be accessing web-based resources without evidence.

## Data availability statement

The raw data supporting the conclusions of this article will be made available by the authors, without undue reservation.

## Ethics statement

The studies involving human participants were reviewed and approved by Betsi Cadwaladr University Health Board NHS Ethics Committee and School of Psychology Ethics Committee, Bangor University. The participants provided their written informed consent to participate in this study.

## Author contributions

DO produced the first draft of the manuscript. JH and MW contributed equally to developing the line of argument and the editing to produce the final manuscript. All authors contributed to the article and approved the submitted version.

## Funding

This work was funded directly by an anonymous donation and coordinated through the Development and Alumna Relations Office, Bangor University. The funder had no involvement in the conduct of the research and/or preparation of the article.

## Conflict of interest

JH is the author of the book for which the intervention is based.

The remaining authors declare that the research was conducted in the absence of any commercial or financial relationships that could be construed as a potential conflict of interest.

## Publisher’s note

All claims expressed in this article are solely those of the authors and do not necessarily represent those of their affiliated organizations, or those of the publisher, the editors and the reviewers. Any product that may be evaluated in this article, or claim that may be made by its manufacturer, is not guaranteed or endorsed by the publisher.
